# Sample size requirements for separating out the effects of combination treatments: Randomised controlled trials of combination therapy vs. standard treatment compared to factorial designs for patients with tuberculous meningitis

**DOI:** 10.1186/1745-6215-12-26

**Published:** 2011-02-02

**Authors:** Marcel Wolbers, Dorothee Heemskerk, Tran Thi Hong Chau, Nguyen Thi Bich Yen, Maxine Caws, Jeremy Farrar, Jeremy Day

**Affiliations:** 1 Hospital for Tropical Diseases, Wellcome Trust Major Overseas Programme and Oxford University Clinical Research Unit, Ho Chi Minh City, Vietnam; 2 Hospital for Tropical Diseases Ho Chi Minh City, Vietnam; 3 Pham Ngoc Thach Hospital, Ho Chi Minh City, Vietnam

## Abstract

**Background:**

In certain diseases clinical experts may judge that the intervention with the best prospects is the addition of two treatments to the standard of care. This can either be tested with a simple randomized trial of combination versus standard treatment or with a 2 × 2 factorial design.

**Methods:**

We compared the two approaches using the design of a new trial in tuberculous meningitis as an example. In that trial the combination of 2 drugs added to standard treatment is assumed to reduce the hazard of death by 30% and the sample size of the combination trial to achieve 80% power is 750 patients. We calculated the power of corresponding factorial designs with one- to sixteen-fold the sample size of the combination trial depending on the contribution of each individual drug to the combination treatment effect and the strength of an interaction between the two.

**Results:**

In the absence of an interaction, an eight-fold increase in sample size for the factorial design as compared to the combination trial is required to get 80% power to jointly detect effects of both drugs if the contribution of the less potent treatment to the total effect is at least 35%. An eight-fold sample size increase also provides a power of 76% to detect a qualitative interaction at the one-sided 10% significance level if the individual effects of both drugs are equal. Factorial designs with a lower sample size have a high chance to be underpowered, to show significance of only one drug even if both are equally effective, and to miss important interactions.

**Conclusions:**

Pragmatic combination trials of multiple interventions versus standard therapy are valuable in diseases with a limited patient pool if all interventions test the same treatment concept, it is considered likely that either both or none of the individual interventions are effective, and only moderate drug interactions are suspected. An adequately powered 2 × 2 factorial design to detect effects of individual drugs would require at least 8-fold the sample size of the combination trial.

**Trial registration:**

Current Controlled Trials ISRCTN61649292

## Background

Tuberculous meningitis (TBM) is the most severe form of *M. tuberculosis *infection, and kills or disables more than half of those affected [1]. Effective new intervention strategies are thus urgently needed. The study hypothesis of a new randomized clinical trial whose protocol is published along with the current manuscript [2] is that current anti-mycobacterial regimes are not potent enough and that increasing the levels of effective anti-mycobacterial drugs in the cerebrospinal fluid will improve clinical outcome. This hypothesis is tested by simultaneously increasing the dose of rifampicin and adding levofloxacin to the standard treatment and performing a two-group comparison of intensified versus standard treatment. This approach allows testing of the primary study hypothesis but it does not allow quantification of the individual effects of each drug or facilitate exploration of potential synergistic or antagonistic interactions between them.

A 2 × 2 factorial design would potentially answer these questions by simultaneously randomizing patients to one of two levels of factor 1 (e.g. standard treatment vs. standard treatment + treatment A [intensified rifampicin]) and to one of two levels of factor 2 (e.g. standard treatment vs. standard treatment + treatment B [levofloxacin]) such that one quarter of the patients receive each of the possible combination treatments. A typical analysis of factorial designs estimates the overall treatment effect of treatment A by pooling across levels of factor 2, i.e. the estimate corresponds to the average of the treatment effects of treatment A in patients randomized to standard treatment for factor 2 and those randomized to treatment B for factor 2, respectively. Crucially, all randomized patients are included in the estimation of the effect of treatment A and, thus, a 2 × 2 factorial design has essentially the same power as a corresponding simple randomized trial which would only randomize factor 1. Factorial designs thus have the potential to answer two (or multiple) questions for the "price" of one and appear to be ideal designs for evaluating combination treatments.

One major complication of studying combination treatments is that they may interact, i.e. that the effect of treatment A is different depending on whether it is added to standard treatment or standard treatment+treatment B (or, equivalently, that the effect of adding both treatments A and B is not equal to the sum of their individual effects). Both synergistic and antagonistic (negative) interactions are possible. While interactions can be studied in the framework of factorial designs, they complicate their analysis and interpretation. In particular, the interpretation of the treatment effect estimate from the standard analysis outlined above is problematic [3-6].

The need to improve outcomes in diseases with high morbidities and mortalities is clear. In rare diseases, such as TBM, the pace of progress is slowed because of the time needed to recruit sufficient numbers of patients to studies powered to appropriate clinical endpoints. Clinical experts may judge that the intervention with the best prospects to improve outcomes is the addition of a combination of several drugs to the standard of care. This new intervention could either be tested with a simple randomized trial of combination therapy versus standard of care or with a factorial design which evaluates each intervention component separately. The objective of the present manuscript is to compare these approaches in terms of their statistical power using our study in TBM as a representative example. We find that in certain situations performing the pragmatic combination trial is the preferred approach.

## Methods

### Sample size calculation for the new TBM trial

The primary endpoint of our proposed study in TBM is overall survival during a follow-up period of 9 months. We expect a 9-month mortality rate of approximately 40% in the control arm and an absolute risk reduction of 10% (from 40% to 30%) due to intensified combination treatment was judged as both realistic and clinically relevant. Assuming proportional hazards, these mortality estimates translate into a hazard ratio of 0.7 [= log(1-0.3)/log(1-0.4)], i.e. a 30% risk reduction due to intensified treatment on the hazard ratio scale. Using Schoenfeld's formula [7], a total of 247 deaths are required to detect a hazard ratio of 0.7 based on a two-sided test at the 5% significance level with 80% power; assuming an overall mortality rate of 35% in the trial, this translates into a need to enroll 706 patients. In order to account for potential deviations from our assumptions and losses to follow-up, a safety margin of 6% was added to this number leading to a total sample size of 750 patients (375 per treatment group). Further details of the sample size calculation are described in the study protocol [2].

### Factorial designs without interactions

We first assumed that the combination treatment effect is as described above, i.e. that the hazard ratio of combination treatment versus control is 0.7, and that there is no interaction present, i.e. that the total combination treatment effect (as measured on the log-hazard ratio scale) is equal to the sum of the individual contributions of treatment A [intensified rifampicin] and B [levofloxacin].

For our investigations we varied the total sample size of a hypothetical factorial trial from the size of the two-group trial (i.e. 750 patients) to 8-fold its size (i.e. 6'000 patients) and assumed that the total observed number of deaths per 750 included patients was 247 (as in the sample size calculation above). In addition, we varied the contribution of the more potent of the two individual treatments to the combination treatment effect from 50-100%. The analysis was assumed to be a Cox regression analysis with treatment indicators for each treatment as covariates. We then calculated the statistical power of the following comparisons of the factorial design:

- Probability that the two tests for the effects of individual treatment A and B both reach statistical significance.

- Probability that the test of the more potent of the two treatments reaches statistical significance.

- Probability that at least one of the two tests for individual treatment A and B effects reaches statistical significance.

- Probability that the comparison of combination treatment versus standard of care reaches statistical significance in the factorial design.

- Power of a two-arm trial of combination treatment versus standard of care with the same sample size as the factorial design.

Details regarding the power calculation are provided in Additional file 1: appendix. Of note, the formulas in the appendix are approximations. In addition, we performed a simulation study where we estimated exact power based on results from Cox regression analyses of simulated trial data assuming exponentially distributed survival times and averaging results over 10'000 simulated trials for each parameter setting. As results from this simulation study were qualitatively identical to the approximations, the simulation results are not reported here.

### Factorial designs with interactions

In a second step, we investigated the impact of interactions assuming that the effect of either treatment A or B alone leads to a reduction in the hazard of 0.84, i.e. identical effects of each drug alone and a combination treatment effect corresponding to a hazard ratio of 0.84 0.84 = 0.7 in the absence of an interaction. For our investigations we varied the total sample size of a hypothetical factorial trial from 4-fold the size of the two-group trial (i.e. 3'000 patients) to 16-fold its size (i.e. 12'000 patients) and varied the strength of the interaction effect from -200% to + 200% of the effect of either drug alone. An interaction of -200% corresponds to an effect of combination treatment of zero, i.e. the effect of either drug vanishes when combined with the other, an interaction of size -100% indicates that either drug alone works as well as their combination, whereas an interaction of +200% corresponds to a strongly synergistic interaction where the combination treatment effect is twice the sum of the individual effects of A and B alone. The analysis was assumed to be a Cox regression analysis with treatment indicators for each therapy as covariates plus an interaction term. For each scenario, we calculated the power of the following comparisons:

- Probability that the interaction test reaches statistical significance and probability that the one-sided p-value is ≤ 10% (indicating mild evidence for an interaction).

- Probability that the main effect corresponding to treatment A reaches statistical significance.

- Power of a two-arm trial of combination treatment versus standard of care with the same sample size as the factorial design.

Of note, the main effect of treatment A corresponds to an averaged effect of treatment A when added to either standard of care or standard of care plus treatment B and may be difficult to interpret clinically.

### Other conventions and statistical software

Statistical significance was determined as significance at the one-sided 2.5% significance level. We used one-sided tests in the direction of the true (simulated) effect throughout because power formulas are simpler for the connected rejection areas of one-sided tests. No adjustment for multiple testing was performed. All calculations were performed with the statistical software R version 2.9.1 [8].

## Results

**Figure 1:**
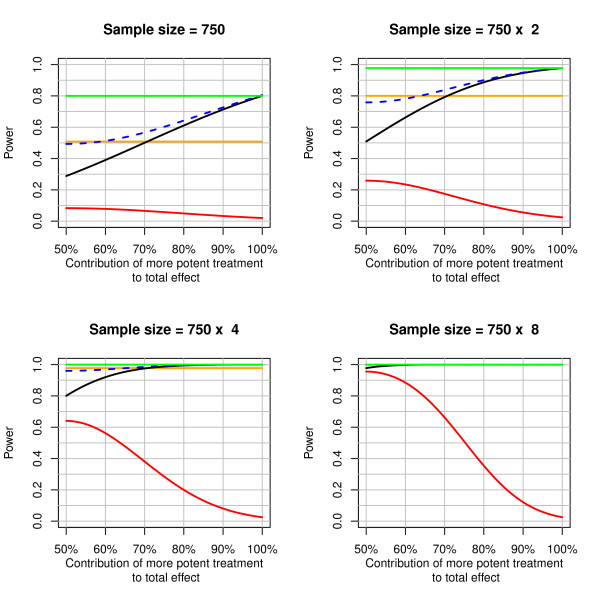
**Power curves for 2 × 2 factorial designs without an interaction assuming a hazard ratio of combination treatment versus standard treatment of 0.7 and an overall mortality of 35%**. Figure legend text: The black, dashed blue, and red lines correspond to the probability that the more potent individual treatment, at least one of the two treatments or both treatments jointly, respectively, reach statistical significance. The orange and green lines correspond to the probability of a significant difference between combination treatment and standard treatment in the factorial trial and in a 2-group trial of combination treatment versus standard treatment with equal sample size, respectively.

Power curves for the factorial design assuming no statistical interactions depending on the sample size and the contribution of the more potent individual treatment are displayed in Figure 1. It is apparent from these curves that if only one drug contributes to the total treatment effect, the power of the factorial design to detect individual treatment effects is essentially equal to the power of the combination treatment trial to detect the combination effect. However, if both drugs contribute to the total effect, power is much diminished. For example, the right lower panel of Figure 1 shows that in order to get 80% power to detect an effect for both individual treatments simultaneously assuming the split-up in effects between the two drugs is between 35:65 to 65:35, one would need an 8-fold increased sample size. Moreover, the figure shows that a factorial trial with suboptimal sample size has a much higher chance of concluding that one of the treatment works than concluding that both jointly work, i.e. that the combination treatment is the optimal treatment, even if both treatments have similar effects. For example, with 1'500 patients and equal effects of both individual treatments, the probability that exactly one of them is significant is 2-times higher than the probability that both jointly reach statistical significance (chances of approximately 50% vs. 25%). Finally, for the comparison of combination treatment versus control, a factorial trial requires twice the sample size of the combination trial for equal power (ignoring multiple testing issues for the factorial design).

**Figure 2:**
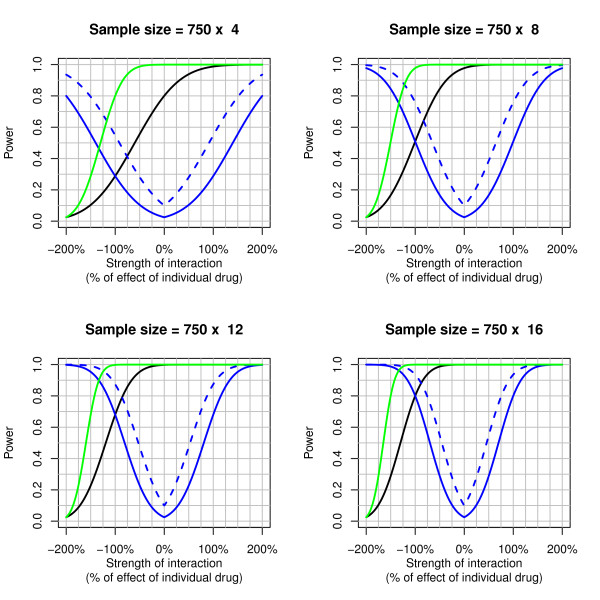
**Power curves for 2 × 2 factorial designs with an interaction assuming equal individual contributions from both treatments corresponding to hazard ratios of 0.84 and an overall mortality of 35%**. Figure legend text: The blue lines correspond to the power of the interaction test (solid line: one-sided 2.5% significance level, dashed line: one-sided 10% significance level), the black line to the power of the main effect for treatment A, and the green line to the power of a 2-group trial of combination treatment versus standard treatment with equal sample size.

Power curves for the factorial design assuming equal individual treatment effects plus an interaction depending on the sample size and the strength of the interaction are displayed in Figure 2. The curves show that the power of the main effect of the trial is strongly reduced in the presence of a negative interaction. Further, in order to detect an interaction of -100%, i.e. that the effect of combination treatment is equal to either drug alone, with 80% power, one would need 16-fold the sample size of the two-group combination trial. With an 8-fold sample size and a liberal one-sided significance level of 10%, power to detect an interaction of this size would be 76%.

## Discussion

We found that a 2 × 2 factorial design powered to detect individual treatment effects would require at least 8-fold the sample size of a two-group combination treatment trial based on the same assumptions. This increase in sample size would guarantee sufficient power to detect both individual treatment effects assuming that the contribution of each individual treatment to the joint effect is at least 35% and that there are no interactions. Moreover, at least an 8-fold increased sample size is required to detect at least mild evidence for a qualitative interaction of -100%, i.e. that either treatment alone has the same effect as the combination. The reasons for this high price for separating out combination effects come from two main sources: First, if there is no interaction and both drugs have equal contributions to the total effect, their individual effect would be half the combination effect which requires 4-fold the sample size to detect individually (and more than 5-fold the original sample size to detect both jointly with 80% power). A further increase in sample size is mandated to protect against the possibility of unequal contributions of the two drugs. Second, it is well-known that interaction tests lack power, i.e. in order to detect an interaction of the same size as a main effect with equal power, 4-fold the sample size is required [3]. Moreover, modest negative interactions can considerably diminish the power to detect treatment effects in the factorial design even in cases that have little power to detect this interaction [4].

An 8-fold sample size increase, i.e. 6'000 patients in total, would transform our study protocol from what will be the largest trial ever conducted in TBM to an impossible study. Thus, a factorial design would have to make much more aggressive assumptions regarding the individual treatment effects in order to arrive at a more realistic sample size. However, such assumptions would lead to an increased likelihood that the trial is underpowered. Even if either of the two intervention effects reaches significance in such an underpowered study, we have shown that chances are high that substantial interaction effects are not discovered and that only one of the treatments reaches statistical significance even if both are equally effective. An earlier publication showed that typical analysis strategies in factorial designs have a relatively low chance of finding the optimal treatment combination in many situations [5] and these chances are even more diminished if the trial does not even have sufficient power to detect the individual treatment effects.

Based on these findings, we believe that a pragmatic two arm trial of combination therapy versus standard treatment is the design of choice under the following assumptions: First, an adequately powered factorial design which allows separating out individual treatment effects is not feasible due to excessive sample size. Second, both interventions test the same broad study hypothesis and the combination is considered most promising. Third, it is considered likely that either both or neither of the two drugs are effective and at most moderate interactions between the two interventions are expected. As we have seen, in case only one of the two interventions is efficacious and there is no interaction, a factorial trial would be optimal and could indeed deliver two answers for the price of one. Factorial designs and, more generally, fractional factorial designs are very efficient designs to screen several potential interventions many of which are likely inefficacious [9]. Fourth, neither of the two interventions has substantially higher costs or is expected to be much more toxic than the other. Finally, a pragmatic combination trial is unlikely to be acceptable for regulatory drug approval which requires proof of efficacy for each individual component.

Our proposed trial in TBM fulfils all of the above conditions. TBM is a relatively rare disease with a limited number of patients globally, but devastating for those who are affected. Both interventions in our TBM trial test the same broad study hypothesis, i.e. that increasing levels of effective anti-mycobacterial drugs in the cerebrospinal fluid will improve treatment outcome. If this trial is successful, it will likely lead to follow-up trials which may further optimize the anti-tuberculosis treatment. This optimization may be based on the new drugs which are currently under development, e.g. TMC207 or PA824. One could in principle also revisit the question whether both components of the intensified treatment are necessary, perhaps based on a 3-arm non-inferiority trial of individual interventions versus combination therapy. However, such a series of simple (superiority and non-inferiority) trials would likely be less efficient than one large factorial trial. If the combination trial is not successful, follow-up trials may focus on new study hypotheses. For example, it could be that drugs which prevent or reduce the risk of infarction result in lower mortality and better outcome for TBM patients [10].

Our main interest in the factorial design is that it allows separating out individual treatment effects and investigation of interactions. An alternative would be to perform the same trial but analyze it as a 4-arm trial instead which focuses on the comparison of each intervention and combination treatment, respectively, to the standard treatment. As we saw, such a trial would only require 2-fold the sample size of the combination trial to detect a combination effect if no adjustment for multiplicity is performed (and 2.7-fold the sample size using a Bonferroni correction). In addition, such a trial could be adaptive, i.e. allow for intermediate dropping of inefficacious arms [11]. However, such an analysis would not exploit the factorial design and have very low power to detect individual treatment effects. Other alternative designs might also be considered. Options would include 3-arm trials of the standard treatment versus the two most promising combinations or to first initiate a pilot study to further assess the safety and pharmacological profile of both interventions and their interaction. However, none of these trials can avoid the fundamental problem that the cost of separating out combination treatment effects into their components may be prohibitively high.

## Conclusions

Pragmatic combination trials of multiple interventions versus standard therapy are valuable in diseases with a limited patient pool if all interventions test the same treatment concept, if it is considered likely that a combination effect is based on contributions from all individual interventions, and only moderate (negative or positive) treatment interactions are suspected. In the case of two interventions, a 2 × 2 factorial design which is adequately powered to detect individual treatment effects would require at least 8-fold the sample size of the combination trial.

## Abbreviations

TBM: tuberculous meningitis

## Competing interests

The authors declare that they have no competing interests.

## Authors' contributions

All authors contributed to the design of the TBM trial protocol and discussions regarding alternative designs which formed the basis for this manuscript. MW performed all statistical calculations and drafted the first version of the manuscript. All authors reviewed the manuscript critically and read and approved the final version.

## Supplementary Material

Additional file 1 **Technical appendix**. Power calculation for 2 × 2 factorial trials. Click here for file
