# Supplementary material for: Sample size requirements for separating out the effects of combination treatments: Randomised controlled trials of combination therapy vs. standard treatment compared to factorial designs for patients with tuberculous meningitis
Source: Trials. 2011 Feb 2;12:26. doi: 10.1186/1745-6215-12-26 (PMC3040154; doi:10.1186/1745-6215-12-26)
Supplement: Additional file 1 — Technical appendix. Power calculation for 2 × 2 factorial trials. [file 1745-6215-12-26-S1.DOC]

## **Technical appendix: Power calculation for 2x2 factorial trials**

We briefly summarize power formulas for 2x2 factorial designs for continuous, binary, and survival outcomes. The exposition is based on the facts stated in Simon and Freedman [6].

It is convenient to code the levels of the factors by -1 and +1 such that the linear predictor of a 2x2 factorial model with an interaction has the form β_0_+ β_1_x_1_+ β_2_x_2_+ β_3_x_1_x_2_ where the covariates x_1_ and x_2_ correspond to the factor levels of factor 1 and 2, respectively. With this coding, the main effects plus the interaction are directly related to the estimated regression parameters, see table A1 below. For survival analyses based on Cox regression, the intercept β_0_ would evidently be omitted.

Approximate variances of parameter estimates based on this model assuming a balanced design (i.e. equal sample size for each of the 4 treatment combinations) are displayed in table A1 below. Moreover, the estimates of the main effects and the interaction are approximately independent of each other (and exactly independent in case of a linear model with continuous outcomes).

Table A1: Approximate variances of parameter estimates in the 2x2 factorial design.

|  |  | Approximate variance of parameter estimates | | |
| --- | --- | --- | --- | --- |
|  | Para-meter | Continuous outcome | Binary outcome  (logistic regression) | Survival outcome  (Cox regression) |
| Main effect for treatment 1 | 2β_1_ | 4σ^2^/N | 4/(Np(1-p)) | 4/D |
| Main effect of treatment 2 | 2β_2_ | 4σ^2^/N | 4/(Np(1-p)) | 4/D |
| Interaction | 4β_3_ | 16σ^2^/N | 16/(Np(1-p)) | 16/D |
| Effect of treatment 1 without treatment 2 | 2β_1_-2β_3_ | 8σ^2^/N | 8/(Np(1-p)) | 8/D |
| Effect of treatment 2 without treatment 1 | 2β_2_-2β_3_ | 8σ^2^/N | 8/(Np(1-p)) | 8/D |
| Effect of combining treatments 1 and 2 | 2β_1_+2β_2_ | 8σ^2^/N | 8/(Np(1-p)) | 8/D |

N refers to the total sample size of the trial, σ^2^ to the error variance in a linear model, p to the average probability of a failure
 for the binary outcome and D to the total observed number of events in the trial with a survival outcome. Parameter estimates correspond to log-odds ratios for binary outcomes and log-hazard ratios for survival outcomes.

Based on the target value of an estimate δ and its approximate standard error s.e. from Table A1, the approximate power based on a one-sided test at level α/2 can then be determined as

$$\text{Power}=\Phi\left(\frac{|\delta|}{s.e._{\delta}}-z_{1-\alpha/2}\right)\text{ }$$

where z_1-α/2_ and Φ corresponds to the respective quantile and the cumulative distribution function of the standard normal distribution.

$\text{Power}=\Phi\left(\frac{|\log(0.84)|}{\sqrt{4/494}}-1.96\right)\approx\text{ 0.5.}$As an example, the power of a 2x2 factorial design for a survival outcome with 1500 patients in total and approximately 494 observed deaths to detect a hazard ratio of 0.84 for of the main effect of treatment 1 would be approximately
